# Homeostasis Disrupted and Restored—A Fresh Look at the Mechanism and Treatment of Obesity During COVID-19

**DOI:** 10.3389/fcvm.2021.721956

**Published:** 2021-08-27

**Authors:** Jacqueline Dickey, Camelia Davtyan, David Davtyan, Heinrich Taegtmeyer

**Affiliations:** ^1^McGovern Medical School at The University of Texas Health Science Center at Houston, Houston, TX, United States; ^2^The University of Texas Health Science Center at Houston School of Public Health, Houston, TX, United States; ^3^Department of Internal Medicine, University of California Los Angeles, Los Angeles, CA, United States; ^4^Department of General Surgery, Cedars-Sinai Medical Center, Glendale, CA, United States; ^5^Department of Internal Medicine, Division of Cardiology, McGovern Medical School at The University of Texas Health Science Center at Houston, Houston, TX, United States

**Keywords:** obesity, weight reduction, bariatric surgery, metabolism, COVID-19

## Abstract

The prevalence of obesity in the United States approaches half of the adult population. The COVID-19 pandemic endangers the health of obese individuals. In addition, the metabolic syndrome poses a challenge to the health of obese adults. Bariatric surgery and diet restore metabolic homeostasis in obese individuals; however, it is still unclear which strategy is most effective. For example, intermittent fasting improves insulin sensitivity and diet alone decreases visceral adipose tissue at a disproportionately high rate compared to weight loss. Bariatric surgery causes rapid remission of type 2 diabetes and increases incretins for long-term remission of insulin resistance before meaningful weight loss has occurred. Malabsorptive surgeries have provided insight into the mechanism of altering metabolic parameters, but strong evidence to determine the duration of their effects is yet to be established. When determining the best method of weight loss, metabolic parameters, target weight loss, and risk-benefit analysis must be considered carefully. In this review, we address the pros and cons for the optimal way to restore metabolic homeostasis.

## Key points

- Obesity is a state of metabolic dysregulation, and affects susceptibility to COVID-19 infections.
- Bariatric surgery results in the most sustainable weight loss of known strategies for obesity treatment and causes rapid, persistent improvement in insulin resistance.
- Bariatric surgery has metabolic benefits independent of weight loss, but the exact mechanisms are still unclear.
- Malabsorptive bariatric surgery restores metabolic health to a greater extent than solely restrictive bariatric surgery.
- More knowledge is needed to account for the distinction between bariatric surgery's effect on visceral adipose tissue and subcutaneous adipose tissue.
- Intermittent fasting improves metabolic health by inducing adaptations that respond to periods of fasting and feeding, thereby increasing metabolic flexibility.

**Figure 1:**
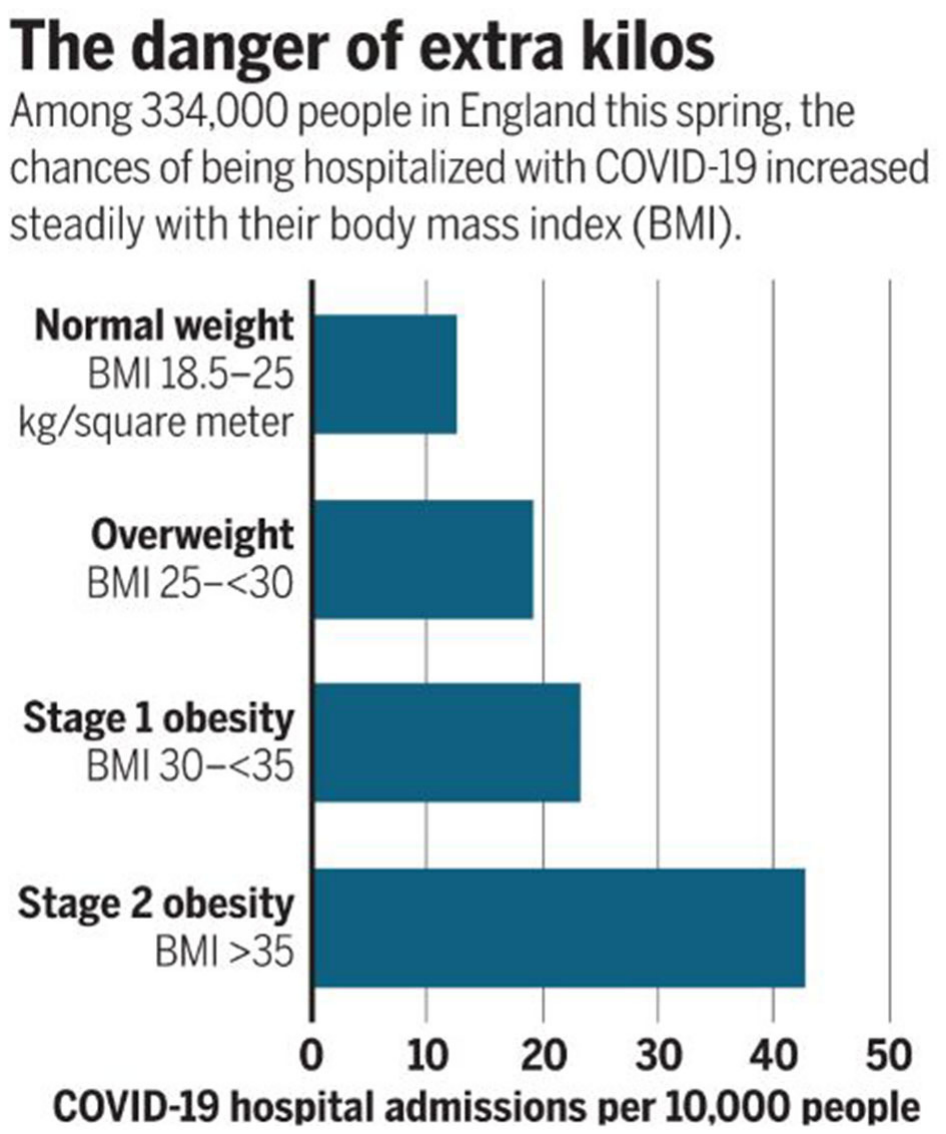
Direct relationship between BMI and risk of severe COVID-19 illness. Data from Hamer et al. reveal an increase from <15 COVID-19 hospital admissions per 10,000 people in patients with normal weight compared to >40 admissions in those with Stage 2 obesity. Reproduced from Wadman with permission (2).

**Figure 2:**
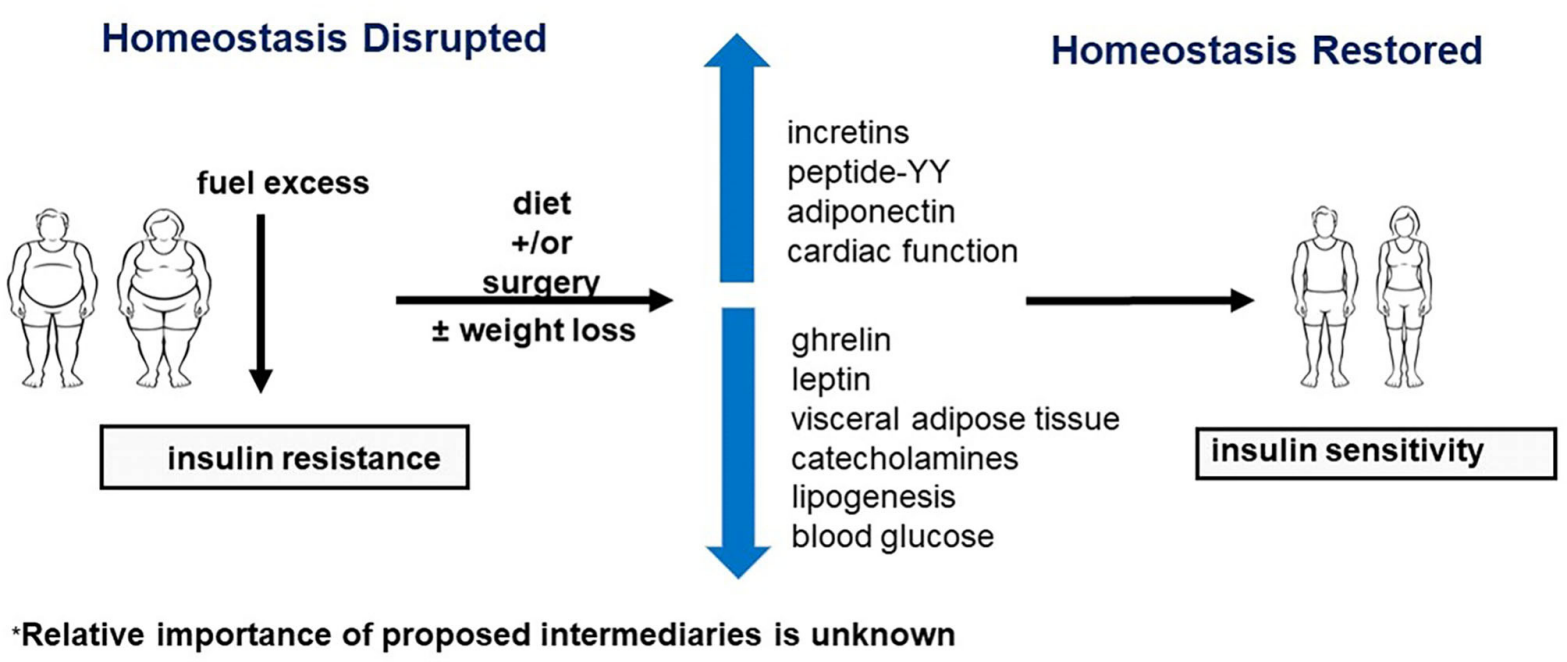
Restoration of homeostasis by neuroendocrine regulation. Surgery and diet may improve insulin sensitivity by affecting various components of the neuroendocrine axis. Refer to text for further discussion.

Obesity is a vexing problem. Despite an abundance of data on the serious health risks of obesity, its prevalence is increasing around the world. In the United States, the prevalence of obesity (defined by BMI of 30–39.9 kg/m^2^) is currently 42.4%, and the prevalence of severe obesity (defined by BMI >40 kg/m^2^) is 9.2% (1). In addition to adverse effects on people's well-being, the costs for the treatment of obesity and related comorbidities are formidable. More importantly, in the last year obesity has also been identified as a major risk factor for susceptibility to severe COVID-19 disease (Figure 1). Obesity may also decrease efficacy of the COVID-19 vaccine (2, 3) Here, we discuss obesity as a process of disrupted fuel homeostasis, and the treatment of obesity as an attempt to restore this homeostasis and possibly also lower susceptibility to severe COID-19 infections (Figure 2).

The long-term success rates of weight loss vary considerably depending on the treatment strategy (4). Historically, diet and exercise were the mainstay of obesity treatment. Pharmacotherapy was subsequently introduced without much success, while bariatric surgery was offered to patients with a BMI > 40 kg/m^2^ or those with comorbidities and a BMI > 30–35 kg/m^2^, depending on the surgical method.

Surgical procedures include Roux-en-Y gastric bypass surgery, sleeve gastrectomy, adjustable gastric banding, and the less commonly used biliopancreatic diversion (5). Many studies have already investigated metabolic outcomes of bariatric surgery, but study protocols in the existing literature are heterogeneous. Consequently, meta-analyses are difficult to perform due to the inconsistencies between different studies.

More work is needed to address this important public health issue, including a standardized comparison of the metabolic consequences of various bariatric surgery options, as well as diet and pharmacotherapy. As rigorously controlled comparative data begin to emerge, a fresh look at obesity and its treatment may offer a new perspective.

## Disrupted Fuel Homeostasis

### The Metabolic Syndrome and COVID-19

Metabolic syndrome is defined by an increase in any three of the following parameters: waist circumference, blood pressure, blood glucose level, or triglyceride level, or high-density lipoprotein cholesterol concentration (6). The interplay among these features is difficult to sort out, as some are seen in one patient but not the next. However, in all the leading theories, insulin resistance is a core component. Metabolic syndrome is particularly important in the context of COVID-19, as it causes a three times higher chance of death, and a four to five times higher probability of invasive mechanical ventilation, acute respiratory distress syndrome, and admission to the intensive care unit. Obesity may also impair an immune response to the COVID-19 vaccine (3, 7). In addition, compared to COVID-19 patients with normal glucose or with diabetes, those with hyperglycemia at hospital admission had higher mortality and lower PaO2/FiO2 (8).

To address the significance of insulin resistance, we discuss a recent study by Yoshino et al., in which the authors concluded that weight loss, whether induced by bariatric surgery, or by strict dieting alone, was key to restoring insulin sensitivity (9). In this matched prospective cohort study, obese individuals with type 2 diabetes underwent either Roux-en-Y gastric bypass or were placed on a diet to achieve 16–24% weight loss. Weight loss in both groups increased hepatic insulin sensitivity as indicated by increased suppression of glucose production and increased glucose disposal, with no significant difference between groups (9). The report by Yoshino et al. suggests, at first glance, that the mechanism of weight loss does not impact the outcomes concerning insulin sensitivity. However, this study has considerable limitations, and the results must be interpreted with caution. As the authors mentioned, the subjects were not randomized, and the sample size may have been inadequate. We also noted that two-thirds of the participants were women, whose metabolic profiles differ from men with respect to lipid metabolism, and to their predisposition for central obesity. Most importantly, both groups, the surgical and the non-surgical group, were placed on diets to achieve weight loss, suggesting that the lack of between-group differences may be due to diet and weight loss combined. Several studies also suggest that various metabolic improvements, such as decreased visceral adipose tissue, increased insulin sensitivity, and increased levels of high-density lipoprotein cholesterol occur in the absence of weight loss (10). Given these confounders, it is difficult to determine whether other, more important, factors are at play.

Another confusing component of disrupted fuel homeostasis in metabolic syndrome is that there are two groups of outliers in the general population: “metabolically healthy” obese individuals and “metabolically obese” normal weight individuals. Metabolically obese normal weight individuals (24% of normal weight individuals) are primarily a result of genetics and lifestyle factors, whereas metabolically healthy obese individuals (32% of obese individuals) are impacted by a multitude of factors, such as inflammation of adipose tissue (11, 12). In both groups, poor metabolic “fitness” is strongly correlated with increased hepatic fat stores, which are likely a result of de novo lipid synthesis, increased uptake, and decreased disposal (13). These individuals with disrupted fuel homeostasis complicate the hypothesis that weight loss alone is the mechanism of restoring insulin sensitivity (14).

Additionally, considerable differences in metabolic risk factors in obese individuals (BMI 30–40 kg/m^2^) exist, depending primarily on ectopic fat and lifestyle factors, such as diet and exercise (4, 13). In a landmark paper by Reaven et al., the authors noted an association between insulin resistance and upregulation of the sympathetic nervous system, even suggesting a potential causal relationship (6). An increased adrenergic response may be the mechanism behind other components of metabolic syndrome, such as increased non-estrified long-chain fatty acids (FAs) in the plasma and arterial hypertension. Indeed, recent data have demonstrated a bidirectional relationship of leptin with the sympathetic nervous system and thus, energy storage and mobilization (15). Furthermore, lifestyle changes which decrease insulin resistance and sympathetic nervous system activity, including exercise and caloric control, appear to protect against the metabolic syndrome. It is not proven, however, that these lifestyle modifications are exclusively responsible for the mechanism to restore metabolic normalcy, and the genetic profile likely plays a role as well (6, 12).

## Restoring Fuel Homeostasis

### Metabolic Impacts of Diet and Calorie Restriction

#### Metabolic Impact: Insulin Resistance

To understand which metabolic improvements to attribute to weight loss, diet, or surgery, it is helpful to consider how diet impacts insulin resistance in the absence of significant weight loss. De Cabo et al. established that a 2.5% weight loss caused by 22 days of fasting every other day resulted in a disproportionately high (57%) decrease in fasting insulin levels (16). In another study, individuals not undergoing surgery showed improvements in insulin sensitivity and beta-cell function similar to operated individuals both following similar fasting protocols. These results suggest that fasting may be responsible for the acute metabolic improvements in the surgery group (4).

#### Metabolic Impact: Visceral and Ectopic Excess Body Fat

Another parameter followed by the research work in obesity is the assessment of visceral adipose tissue (VAT), estimated by waist circumference and BMI taken together. Increased VAT, regardless of BMI, is associated with increased cardiovascular and metabolic risk (4). Similarly, excess body fat is highly associated with poor metabolic health and is a far better predictor of metabolic health than BMI (13). The accumulation of visceral and ectopic fat represents disrupted lipid homeostasis due to excessive fuel intake, excessive fuel storage, or deficient fuel breakdown. The amount of visual adipose tissue is readily decreased and insulin sensitivity is readily restored at 3 months after bariatric surgery (17). In contrast there is no effect on liposuction on insulin action with evidence for fatty liver disease (18, 19).

#### Diet: Intermittent Fasting

Intermittent fasting is an increasingly popular strategy for obesity. Intriguingly, after 6 months, women who followed an intermittent fasting protocol of 5 days of regular diet and 2 days of significant calorie restriction per week had a greater decrease in waist circumference than women who just reduced caloric intake by 25% every day (16). Hence, it is reasonable to assume that intermittent fasting per se improves metabolic abnormalities, including insulin resistance.

Supportive evidence suggests improved insulin sensitivity with intermittent fasting in a randomized crossover study that supervised standardized meal consumption in prediabetic men over a five-week period (20). In this study, food intake was matched in both arms with the goal of maintaining current weight. Compared to the matched group that consumed meals over a 12-h period, subjects consuming the same amount of calories over 6-h period, mean postprandial insulin levels were significantly lower (26.9 mU/l) (20). This proof-of-concept study provided the first evidence for the effectiveness of early time-restricted feeding (eTRF) which stresses eating early in the day in alignment with circadian rhythms of metabolism (21). The improvements in insulin levels persisted during the 7-week washout period, indicating that a fasting protocol may be necessary in the diet-only group before one can draw firm conclusions in the Yoshino et al. study (9).

Another important factor is the duration of follow-up. In one 12-month study, insulin sensitivity and lipid levels failed to improve despite weight loss from bariatric surgery (22). Other studies have reported improved insulin and lipid levels after weight loss, indicating that results may differ when taken in an acute vs. long-term setting (9). Although it is tempting to deduce that weight loss accounted for improved insulin sensitivity, it seems that fasting protocol, diet, and time since surgery are potential confounders.

### A Comparison of Bariatric Surgeries

The most common bariatric surgery procedures performed in the United States are currently sleeve gastrectomy (61%), and RYGB (17%), with LAGB and biliopancreatic diversion (2% together) falling out of favor (23). Novel gastrointestinal surgeries that target diabetes, such as duodenal-jejunal bypass and ileal interposition, provide helpful clues to the mechanism of restored insulin sensitivity following bariatric surgery (5). Briefly, RYGB excludes most of the stomach just distal to the gastroesophageal junction (GEJ). It bypasses the entire duodenum and part of the jejunum as the 30 cc stomach pouch attached to the esophagus is anastomosed to the distal jejunum. Sleeve gastrectomy removes a significant portion (60–80%) of the stomach along the greater curvature, and adjustable gastric banding is an inflatable/adjustable silicone band placed around the superior portion of the stomach, just distal to the GEJ. Biliopancreatic diversion removes the stomach just as in gastric sleeve, which is anastomosed to the distal portion of the ileum. The excluded duodenum, jejunum, and proximal ileum are anastomosed to the alimentary limb, allowing biliary and pancreatic secretions to mix with ingested nutrients (5).

The current standard of care in the United States is to allow the patient to choose among these procedures, and patients may find the following information helpful in the decision (Table 1) (23, 24). After 5 years, 35% of LAGB patients had failed to lose weight compared to 4% of RYGB patients, and metabolic parameters were inferior in LAGB patients after 2 years (25). In general, bypass surgeries have caused more durable weight loss and better insulin control than restrictive surgeries, or than lifestyle management (5, 23). Long-term improvements in dyslipidemia and hypertension have been less consistent across studies. Of note, RYGB has higher complication rates than sleeve gastrectomy, including a higher rate of reoperation, perioperative mortality, and readmission (23). However, overall perioperative mortality remains low with RYGB (around 0.2%), and adverse events occur in 1–9% of patients undergoing RYGB (23).

**Table 1:** Malabsorptive surgeries cause more dramatic weight loss and metabolic impacts than purely restrictive surgeries.

**Surgery type**	**Malabsorptive**	**Restrictive**
Sleeve gastrectomy		X
RYGB	X	X
LAGB		X
Biliopancreatic diversion	X	X
Duodenal-jejunal bypass	X	
Ileal interposition	X	

### Metabolic Benefit of Bariatric Surgery Independent of Weight Loss

There are several findings that indicate that bariatric surgery has benefits independent of weight loss. There is a rapid resolution of type 2 diabetes within days following bariatric surgery that persists at one and five years (4, 5). Another important finding is a remarkably higher rate of type 2 diabetes remission at 2 years with malabsorptive RYGB (72%) compared to restrictive LAGB (17%) in patients who had lost approximately 30% of their body weight (26).

In addition to remission of diabetes, subjects in a non-randomized, prospective cohort study showed decreased glucose, insulin, and leptin levels within 3 months of surgery, and glucagon-like peptide 1 (GLP-1), glucose-sensitive insulinotropic peptide (GIP), peptide-YY, while pancreatic polypeptide concentrations increased within days (4, 5, 24), On the contrary, fasting did not affect high molecular weight adiponectin, ghrelin, leptin, or GLP-1, suggesting unique effects of surgery compared to diet (20). One explanation for the rapid change in disrupted fuel homeostasis may be that surgery excises or interrupts regions of the digestive system involved in some of the pathologic adaptations of fuel excess. Additional support for these unique benefits of surgery include the nearly identical skeletal muscle gene expression of diabetogenic hormones, such as stearoyl-CoA desaturase (SCD), pyruvate dehydrogenase kinase 4 (PDK-4), and peroxisome proliferator-activated receptor alpha (PPAR-α) among participants who underwent LAGB vs. RYGB despite significant variation in weight loss between the two groups (222% greater mean weight loss at 9 months in RYGB participants compared to LAGB participants) (24). However, lengthening of the intestinal bypass by RYGB does not affect GLP-1 secretion, which suggests that the GLP-1 response after RYGB may not require delivery of nutrients to more distal intestinal segments (27). It is not known whether the metabolic effects of RYGB persist long-term, but the duration of surgery's impact may provide clues as to the mechanism of restored fuel homeostasis. For example, return of a disruption in fuel homeostasis over time may suggest that surgery merely interrupts pathologic metabolic adaptations, whereas permanent improvement in metabolic health may indicate that surgery removes an element of the metabolic pathway essential to disrupted fuel homeostasis.

### Intermediate Steps Between Disruption and Restoration of Metabolic Homeostasis From Diet and Surgery

Given these important mediators, we postulate that there are intermediate steps between diet and surgery and increased insulin sensitivity (Figure 2). These interposed steps may include incretins, appetite-suppressing hormones, known metabolic risk factors, such as VAT, catecholamines, and unknown upper gastrointestinal effects that impact the regulation of blood glucose and lipogenesis. A possible mechanism for these hormonal changes may be related to the impressive reduction of oil-red-O staining in skeletal muscle post-operatively, which may occur in other organs, dramatically impacting the neuroendocrine signaling between adipose tissue and other organs (24). These findings also support the observation that ectopic fat, such as in skeletal muscle, is a key component of global metabolic dysfunction throughout the body (13).

Additional mechanisms may include surgical removal of the gastric fundus with sleeve gastrectomy, which is the site of ghrelin secretion, or hyperstimulation of appetite-suppressing hormone production by increased nutrient delivery to distal portions of the gastrointestinal tract, such as with RYGB. Antidiabetic effects are also observed in patients with decreased nutrient delivery to the upper gastrointestinal tract, possibly due to anti-incretin factors released from the proximal bowel (4, 26, 28).

### Return to Normalcy in the Cardiovascular System After Surgery-Induced Weight Loss

Obesity affects the cardiovascular system at many different levels. In one study, 42% of obese participants had left ventricular diastolic dysfunction on tissue Doppler imaging (14, 24). Compared to non-obese patients with heart failure with preserved ejection fraction (HFpEF), obese patients with HFpEF had greater volume overload and right ventricular dysfunction, and decreased levels of brain natriuretic peptide and cardiac efficiency (4, 29). Obese patients also have an approximately 50% higher prevalence of atrial fibrillation than normal-weight patients, but the reversibility of arrhythmias following bariatric surgery is still unknown (4). These obesity-related abnormalities may be related to cardiac fibrosis, hypertrophy, and impaired microvascular coronary perfusion (in hearts of diabetic patients), and the cardiovascular impact of COVID-19 has the potential to further compound these problems (5).

Given the detrimental cardiac effects of obesity, pharmacologic agents have been tested as treatment options without success. We have previously proposed that obesity related cardiac dysfunction is likely a result of fuel excess, which results in production of reactive oxygen species (ROS). The overwhelmed heart may protect itself from further damage with insulin resistance in response to ROS. As a result, abruptly inducing insulin sensitivity overrides this protective response, resulting in cytotoxic damage (30). Worsening cardiac dysfunction with thiazolidinedione (TZD) use in a diabetic heart supports this proposition, as TZDs contribute to fuel overload by increasing glucose uptake and oxidation. Alternatively, metformin has protective effects on the heart by enhancing peripheral glucose oxidation and decreasing FAs (31).

In this context, it is important to emphasize that bariatric surgery-induced weight loss is a feasible solution to improve metabolic abnormalities, as well as cardiac function. Surgery decreased the incidence of major adverse cardiovascular events by more than 40% among a matched cohort of 2,600 obese patients with cardiac disease and by more than 55% among those with heart failure (32, 33). Surgery also decreases the use of antihypertensive drugs (33%), and diet induces a 0.43 mmHg decrease in systolic blood pressure per 1% decrease in body mass index (10, 20, 24). Interestingly, blood pressure is one of the first parameters to change with exercise before any meaningful weight loss occurs (10). Tissue Doppler diastolic velocity also increased by 1.9 cm/sec (95% CI 0.52–3.4) and 1.2 cm/sec (95% CI 0.32–2.1) at 3 and 9 months, respectively, along with improved septal mitral annular velocity (24). Lastly, left ventricular mass decreased linearly for 2 years post-operatively even as weight loss plateaued (14). Correction of metabolism by non-pharmacologic means is the most beneficial way to restore cardiac function.

## Considering Weight Loss: Diet VS. Surgery

Bariatric surgery is significantly more efficacious and long-lasting for weight loss than lifestyle modifications or medical management, inducing up to 70% excess weight loss (5). Surgery also increases survival by three years compared to obese subjects on standard obesity treatment over a 24-year period (34). Patients with type 2 diabetes and excess VAT appear to selectively lose VAT with exercise irrespective of the amount of weight loss, and patients who enrolled in a lifestyle intervention program following bariatric surgery had increased weight loss (4). In addition, exercise and diet seem to disproportionately boost outcomes in overweight and obese COVID-19 patients and may be beneficial to prevent hyperglycemia in the context of COVID-19 (2). Weight loss independent benefits of exercise, such as translocation of GLUT-4 receptors to the skeletal muscle surface, enhanced FAs oxidation by an exercise-induced increase in the number of mitochondria, and increased high density lipoprotein cholesterol may be accounted for when devising a patient-specific weight loss plan (10). Other considerations include bariatric surgery for medically refractory type 2 diabetes, and diet and exercise as a first line option for normal-weight patients with type 2 diabetes and other metabolic derangements. However, a combination of diet, exercise, and surgery will likely lead to the best outcomes.

## Conclusions

Obesity treatment continues to confound healthcare professionals. More research is needed to establish a mechanism for the restoration of metabolic homeostasis, a process in for which multiple factors are likely to be responsible. Important concepts to consider include the role of neuroendocrine interactions between adipose tissue and the brain, the hypothesis that sympathetic nervous system upregulation is associated with insulin resistance and perhaps responsible for metabolic syndrome, and the change in skeletal muscle gene expression and reduced ectopic fat deposition following bariatric surgery (15). Intermittent fasting or eTRF appear to be an effective strategy to improve metabolic health by increasing metabolic flexibility, as if conditioning the body's metabolism to undergo periods of stress. The paradoxically damaging effects of abruptly correcting insulin resistance in a fuel-overloaded heart is also significant. Future studies may include a study cohort comprised of a diet and surgery group with matched waist-to-hip ratios and identical fasting protocols or an animal study (with a sham-operated control group) to assess the impact of weight loss on insulin resistance. Additional long-term research is also needed to understand obesity-induced cardiac dysfunction. Gradual weight loss in a time of COVID-19 may decrease morbidity and mortality at a relatively high rate in obese patients, and bariatric surgery should not be postponed (2). Determining long-term outcomes and solidifying our understanding of mechanisms will allow patients and physicians to choose rationally among available weight loss options to restore metabolic health in the patient with disrupted fuel homeostasis. It still remains unknown whether restoring fuel homeostasis also reduces susceptibility to COVID-19 infections. But it seems likely.

## Author Contributions

HT and JD: conceived, designed, and wrote the manuscript. CD and DD: provided critical comments, suggestions, and text. All authors contributed to the article and approved the submitted version.

## Conflict of Interest

The authors declare that the research was conducted in the absence of any commercial or financial relationships that could be construed as a potential conflict of interest.

## Publisher's Note

All claims expressed in this article are solely those of the authors and do not necessarily represent those of their affiliated organizations, or those of the publisher, the editors and the reviewers. Any product that may be evaluated in this article, or claim that may be made by its manufacturer, is not guaranteed or endorsed by the publisher.
